# Cell penetration and chondrogenic differentiation of human adipose derived stem cells on 3D scaffold

**DOI:** 10.2144/fsoa-2021-0040

**Published:** 2021-06-10

**Authors:** Rizka Musdalifah Amsar, Anggraini Barlian, Hermawan Judawisastra, Untung Ari Wibowo, Karina Karina

**Affiliations:** 1 School of Life Science & Technology, Institute of Technology Bandung, Bandung, West Java, Indonesia; 2 Faculty of Mechanical & Aerospace of Engineering, Institute of Technology Bandung, Bandung, West Java, Indonesia; 3 Klinik Hayandra, Jakarta, Indonesia

**Keywords:** β-catenin, cyclin D, LAA, N cadherin, PRP, silk fibroin scaffold, type II collagen

## Abstract

The ability of cells to penetrate the scaffold and differentiate into chondrocyte is important in cartilage engineering. The aim of this research was to evaluate the use of silk fibroin 3D scaffold in facilitating the growth of stem cell and to study the role of L-ascorbic acid and platelet rich plasma (PRP) in proliferation and differentiation genes. Cell penetration and type II collagen content in the silk fibroin scaffold was analyzed by confocal microscopy. Relative expressions of *CDH2*, *CCND1*, *CTNNB1* and *COL2A1* were analyzed by reverse transcription-quantitative PCR (RT-qPCR). The silk fibroin 3D scaffold could facilitate cell penetration. L-ascorbic acid and PRP increased the expression of *CDH2* and *COL2A1* on the 21st day of treatment while PRP inhibited *CTNNB1* and *CCND1*.

## Lay abstract

Cartilage engineering can be a solution to overcome cartilage damage resulting from accident or degenerative diseases. Cartilage engineering combines cells, scaffolds and biochemical factors to produce functional cartilage. The use of stem cells as a cell source in cartilage engineering is beneficial because they can develop into chondrocytes and produce cartilage. Silk fibroin, a natural-based scaffold, facilitates penetration, growth, and differentiation of stem cells into chondrocytes. Cells-scaffold constructs supplemented with L-ascorbic acid or PRP enhance the chondrocyte extracellular matrix indicating that the construct can be develop into cartilage tissue.

Cartilage is a solid connective tissue that forms organs such as trachea, ears, nose, joints and ribs. Cartilage tissue is difficult to repair whenever it is damaged through accidents, congenital defects or degenerative diseases such as osteoarthritis and spinal degeneration. This is due to the low regenerative power of the cartilage tissue [1]. Damaged cartilage tissue could be repaired by transplantation of cartilage tissue by autograft (from the same person) or allograft (from one person to another). However, these methods have several problems such as limited number of cells available, risk of pneumothorax and hematoma [2], skin flap necrosis [3], infections and postoperation trauma. One alternative solution that could be used to repair cartilage tissue damage is tissue engineering technology.

Tissue engineering technology combines three key components called the tissue engineering triad: cells, biomaterial (scaffolds) and regulatory signals [4]. Since the use of chondrocytes as a cell source suffered a weakness in the limited number of cells available [1], stem cells would be a better alternative for cell source [5,6]. Among the stem cells that could be used in cartilage tissue engineering, adipose-derived stem cells (ADSCs) have an advantage compared with other stem cells. The advantages of ADSCs include the high number of cells that could be isolated [7], they were waste products from liposuction and they had the general characteristics of stem cells such as self-renewal and multipotency. Scaffolds to be used for cartilage tissue engineering must be biocompatible, contain pores, have 3D structures and be able to facilitate the proliferation and migration of cells [8]. Silk fibroin is a natural biomaterial that has a strong structure and has already been applied in medicine as material for medical suture thread. Bhardwaj *et al.* [9] used silk fibroin scaffold mixed with chitosan for cartilage tissue engineering and Zeng *et al.* [10] used silk fibroin porous scaffold for nucleus pulposus tissue engineering. Silk fibroin scaffold made with the salt leaching method were biodegradable. According to Wibowo [11], silk fibroin scaffold could be degraded by α-chymotrypsin, collagenase and protease XIV resulting in 19, 12 and 56% mass reduction, respectively. Regulatory signals could come from molecules such as ascorbic acid or by a combination of growth factors like platelet rich plasma (PRP). Both sources had an advantage compared with other substances that were generally used, because both were economical and easy to obtain. Ascorbic acid could increase cell proliferation [12,13], and supported extracellular matrix biosynthesis [14,15]. L-ascorbic acid 2-phosphate is a stable form of ascorbic acid [16] and it had the same role as ascorbic acid in promoting cell proliferation and differentiation. PRP is a blood plasma that contains thrombocyte or blood platelets above the normal concentration in blood in general [17]. Mohammadi *et al.* [18] showed that platelet lysates could support the proliferation and differentiation of mesenchymal stem cells (MSCs) from Wharton’s jelly better than fetal bovine serum (FBS). Activated PRP could secrete different kinds of growth factors that had a role in the proliferation of chondrocytes *in vitro* and in the differentiation of chondrogenic MSCs [19]. The pathway that has an important role in chondrogenesis in the regulation of growth and development of cartilage tissue is the Wnt/β-catenin signaling pathway [20]. In the nucleus, β-catenin regulates genes involved in the process of cell proliferation and differentiation. One of the genes involved in cell proliferation was *CCND1* gene (cyclin D1) and according to Shtutman *et al.* [21], this gene was the target of β-catenin molecules that entered the nucleus. The process of stem cell differentiation into chondrocytes is regulated by SOX9 in the nucleus. SOX9 is a transcription factor for *COL2A1* gene (type II collagen) which is one of the chondrogenic differentiation markers. SOX9 transcription factor is antagonistic with Wnt/β-catenin [22–24]. The activity of Wnt/β-catenin will induce the activation of cyclin D gene so that cell proliferation happens. However, activity of this gene can be suppressed by inhibition of SOX9 toward β-catenin. This would lead the cell to differentiate into a chondrocyte. β-catenin molecules also play a role in cell condensation through the interaction with N-cadherin. N-cadherin is an adhesion molecule that has a role in cell–cell interaction. N-cadherin and N-CAM marks the initiation process of condensation at one of the chondrogenesis steps [25].

Cartilage tissue engineering research using human adipose-derived stem cell (hADSC), silk fibroin scaffold, L-ascorbic acid (LAA) and PRP has already been performed by Barlian *et al.* [26], however, study of cell penetration on the 3D silk fibroin scaffold and the effect of LAA and PRP on the expression of proliferation and differentiation genes during cartilage tissue engineering has not yet been performed. With the scaffold we made, we would like to know whether the cells can penetrate, grow and differentiate toward cartilage. It is important to make sure that the cells can grow and differentiate in our scaffold for its future application. Our hypothesis was that with a pore size 500 μm and the 3D scaffold made of 12% silk fibroin by salt leached method, the hADSCs will penetrate, grow and differentiate well. Herein, we investigated the hADSC penetration in 12% silk fibroin scaffold with 500 μm pore size and effect of 50 μg/ml LAA and 10% PRP on the expression of *CDH2* (N-cadherin), *CTNNB1* (β-catenin), *CCND1* (cyclin D) and *COL2A1* (type II collagen) genes. The level of expression of *COL2A1* gene would be confirmed at the protein level by looking at the presence of type II collagen that are produced by the cells in the silk fibroin scaffold.

## Materials & methods

### Cell culture & expansion

Stem cells that were used in this research came from fat tissue, a waste product from liposuction of patients in Hayandra Clinic (Jakarta, Indonesia). Liposuction was performed by a plastic surgeon. Samples of the fat tissue were given 10% H-remedy recombinant enzyme and incubated in a shaker incubator at 37°C, 300 rpm for 1 h. Low glucose Dulbecco’s Modified Eagle’s Medium (DMEM) (Gibco, Thermo Fisher Scientific, MA, USA) (1 g/l) containing 4 mM L-glutamine was added to inactivate the enzyme and the digested fat tissue was centrifuged at 600 × g for 5 min. The pellet was then added with 10 ml red blood cell lysis solution, incubated for 5 min at room temperature, and centrifuged again at 600 × g for 10 min. The stromal vascular fraction containing stem cells was cultured at 37°C, 5% CO_2_ [27]. The cells were grown until reach 80% confluent. The medium was changed two-times per week. The cells passage 3–4 were used for this experiment.

In this research there were three experimental groups: control; treatment with medium containing LAA; treatment with medium containing PRP. The control medium contained DMEM (Gibco), 1% antimycotic antibiotic (Gibco), and was supplemented with 10% FBS (Gibco). The LAA medium contained DMEM, 1% antimycotic antibiotic, 10% FBS and 1% of 50 μg/ml LAA. The PRP medium contained DMEM, 1% antimycotic antibiotic and 10% PRP. The media were changed every 2 days. All the groups were grown on silk fibroin scaffold with 500 μm pore size.

### Characterization of hADSC

Characterization of the hADSCs was conducted to confirm the cells that were used in this experiment were mesenchymal stem cells (MSCs) based on Dominici *et al.* [28]. Cells passage three were cultured in growth medium containing DMEM Low Glucose (Gibco), Antibiotic-antimycotic (Gibco) and 10% FBS (Gibco). The cells were incubated in 37°, 5% CO_2_. The morphology of the cell was observed with an inverted microscope. After reaching 80% confluence, the cells were harvested using Trypsin-ethylenediaminetetraacetic acid 0.25% (Gibco). The cells were stained with BD STemFlow™ hMSC Analysis Kit according to the manufacture’s protocol. Flourescene-activated cell sorting was performed with MACSQuant Analyzer 10 (Miltenyi Biotec, Bergisch Gladbach, Germany).

For the multipotent assay, cells were cultured in a 24-well plate at seeding density 1 × 10^4^ cell/well. The cells were cultured in growth medium containing DMEM Low Glucose (Gibco), Antibiotic-antimycotic (Gibco) and 10% FBS (Gibco) as control, adipogenic differentiation medium (StemPro^®^ Adipogenesis Differentiation Kit), chondrogenic differentiation medium (StemPro^®^ Chondrogenesis Differentiation Kit) and osteogenic differentiation medium (StemPro^®^ Osteogenesis Differentiation Kit) for 21 days. The replication used for this experiment was triplo. The medium was changed every 2 days. After incubation, the cells were fixed with 4% paraformaldehyde and stained with ‘Alcian Blue’ for chondrocyte’s marker, ‘Alizarin Red’ for osteoblast’s marker and ‘Oil Red O’ for adipocyte’s marker. The cell cultures were washed with phosphate-buffered saline (PBS) and observed with an inverted microscope.

### Scaffold preparation

The preparation of the scaffold was performed as described by Wibowo [11]. Briefly, the 12% w/v scaffold was made using the salt leaching method by dissolving 0.06 g silk fibroin in formic acid containing 12% CaCl_2_ and homogenizing with a stirrer for an hour. Then 2.5 g of 500 μm large NaCl was added to form pores and the suspension was mixed and evaporated in a fume hood overnight. The scaffold was soaked in 70% ethanol for 30 min followed by soaking in distilled water for 3 days, with changing of the water every 6 h to remove any remaining salt. The scaffold was frozen at -80°C for 30 min and cut to a size of 0.5 × 0.5 × 1 mm to be used in this research.

### Cell penetration on the scaffold

Cell penetration analysis was conducted to compare the penetration of the cell into the scaffold on day 7 and 21. The scaffolds were sterilized by autoclaving at 121°C for 15 min. The scaffolds were soaked with DMEM in nontreated 24-well plate and incubated for 2 h at 37°C. The DMEM was poured out and the scaffolds were seeded with 2 × 10^5^ cells in passage four in 20 μl DMEM for each scaffold each well. The seeded scaffolds were incubated at 37°C for 3 h for the cell attachment to the scaffold. To each well 500 μl growth medium (DMEM low glucose, 1% antimycotic antibiotic and 10% FBS) was added and re-incubated at 37°C. The cells were grown for 7 and 21 days with duplication sample replication. The cell-scaffold constructs were fixed with methanol-DMEM series and stained with 4'-6-Diamidino-2-Phenylindole, Dyhidrochloride (DAPI) (Thermo Fisher Scientific) for nucleus staining. The cell-scaffold constructs were observed by Confocal Laser Scanning Microscope (Olympus Fv 1200 [Olympus Global, Tokyo, Japan]). Z-stack slicing was performed by slicing sample (upside and downside) 4 μm per section.

### Gene expression analysis with reverse transcription-quantitative PCR

Total RNAs were isolated from the control, LAA treatment and PRP treatment using SV Total RNA Isolation System (Promega, WI, USA). The concentration and purity of the RNAs were measured using NanoDropTM One/OneC Microvolume UV-Vis Spectrophotometer. cDNAs were synthesized from 500 ng total RNAs using GoScriptTM Reverse Transcription System in accordance with the manual of the kit. RT-qPCR was performed to determine the relative expression of *CDH2, COL2A1, CCND1*, *CTNNB1* genes using GoTaq^®^ 2-Step RT-qPCR System from Promega. The sequence of primers for *COL2A1* genes was taken from previous study [39]. The other primers used in this research were designed using NCBI primer3 apps and their specificity were analyzed with NCBI Primer-BLAST apps. Both apps were present at https://www.ncbi.nlm.nih.gov/tools/primer-blast. The quality of the primers was analyzed with OligoAnalizer apps from https://sg.idtdna.com/calc/analyzer. Primers were ordered from Macrogen (Seoul, South Korea). The list of primers used are shown in Supplementary Table 1. Gene expression was measured using qPCR Biorad CFX 96. The Cq values were normalized with the expression of GAPDH gene and the relative gene expressions were calculated with the 2^-ΔΔCq^ method.

### Presence of the type II collagen matrix

The presence of the type II collagen matrix was analyzed by immunocytochemistry with three replications for each sample. Cells were seeded in scaffold at seeding density 2 × 10^5^ cells/scaffold, incubated in 37°, 5% CO_2_ for 3 h for cell attachment. After incubation, the medium for control, LAA treatment and PRP treatment was added. The cells were cultured for 7 and 21 days and the medium was replaced every 2 days. For immunocytochemistry analysis, the cells were fixed with a methanol-DMEM series. Cells on the scaffolds were permeabilized with 0.05% Tween-20 in PBS and blocked with 3% bovine serum albumin (BSA) in PBS. The seeded scaffolds were incubated with primary antibody (Rabbit Anti-Collagen II Antibody, ab34712, Abcam, UK) in a water bath at 37°C overnight. The samples were washed with PBS followed by incubating in the secondary antibody (Goat Anti-Rabbit IgG H&L Alexa Flour 488, ab150077, Abcam) for 120 min at room temperature. Counterstaining was performed by incubating the seeded scaffolds with DAPI (ThermoFisher) for 10 min. The seeded scaffolds were washed with PBS, then visualized with Confocal Laser Scanning Microscope (Olympus Fv 1200). The image was taken in three microscope fields of view and each of them was projected as a Z stack with a depth of 327 and 4 μm per slice. Semi-quantitative analysis of fluorescence images was calculated using Flowview and Fiji image software.

## Results

### Cell characterization

**Figure 1.**
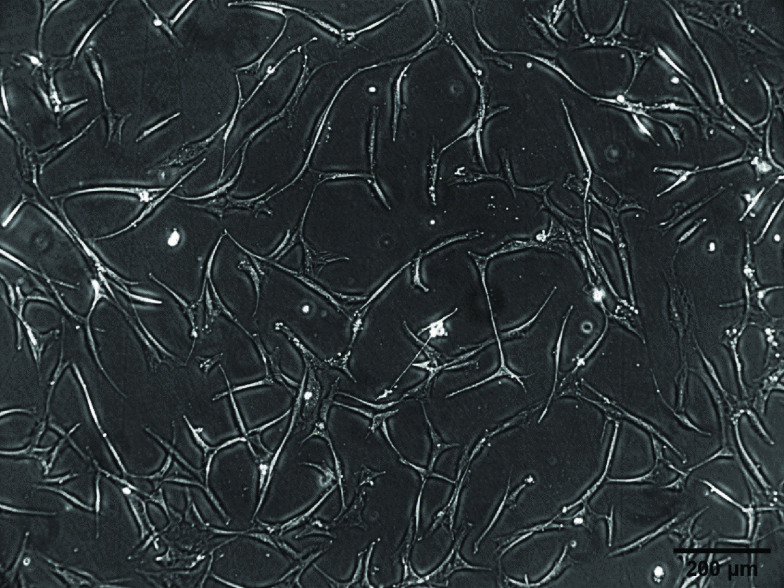
Morphology of human adipose-derived stem cells cultured on polystyrene.

**Figure 2.**
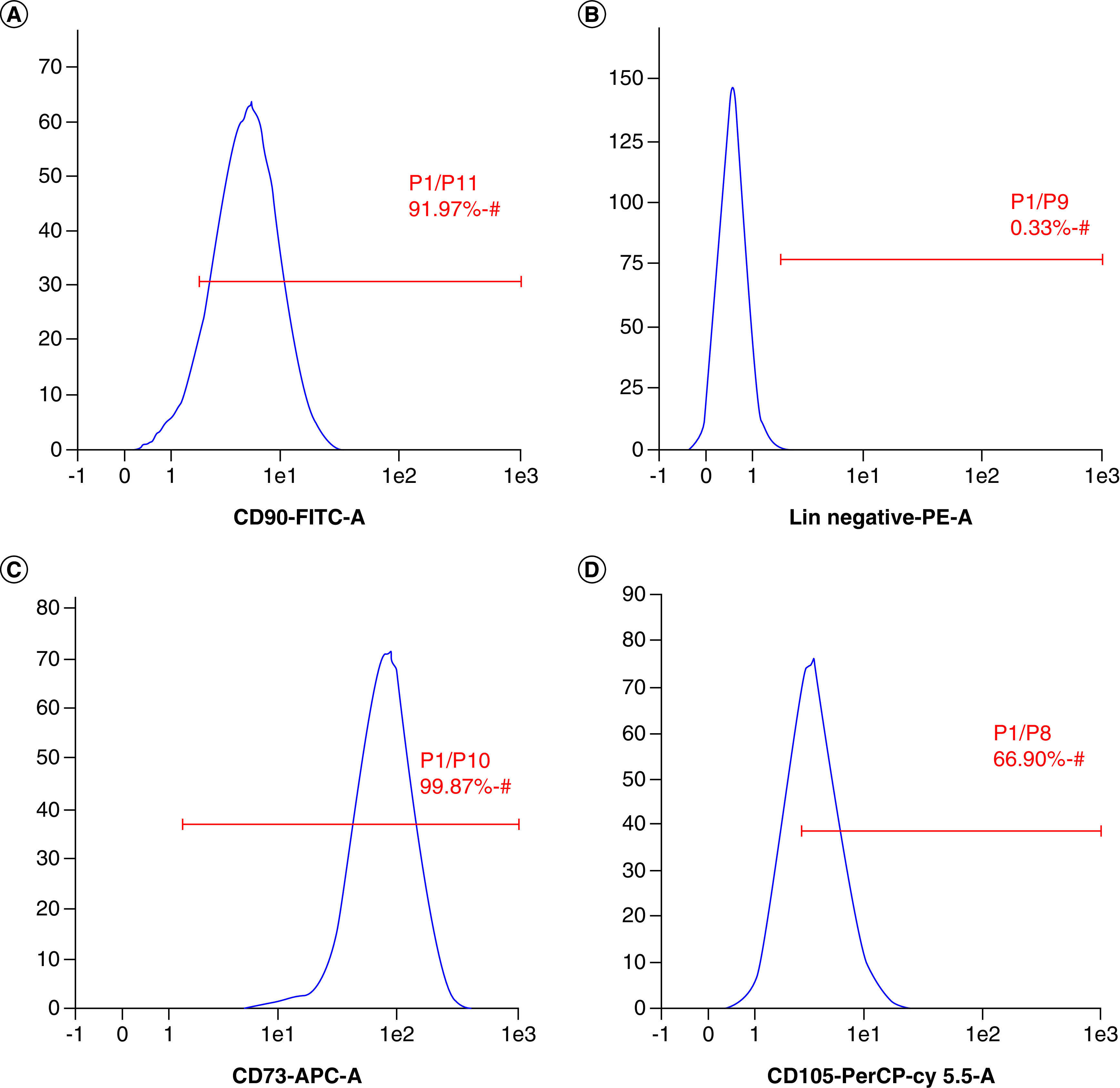
Flow cytometry analysis of human adipose-derived stem cells. **(A)** Surface expressions of CD90. **(B)** Negative markers. **(C)** CD73. **(D)** CD105.

**Figure 3.**
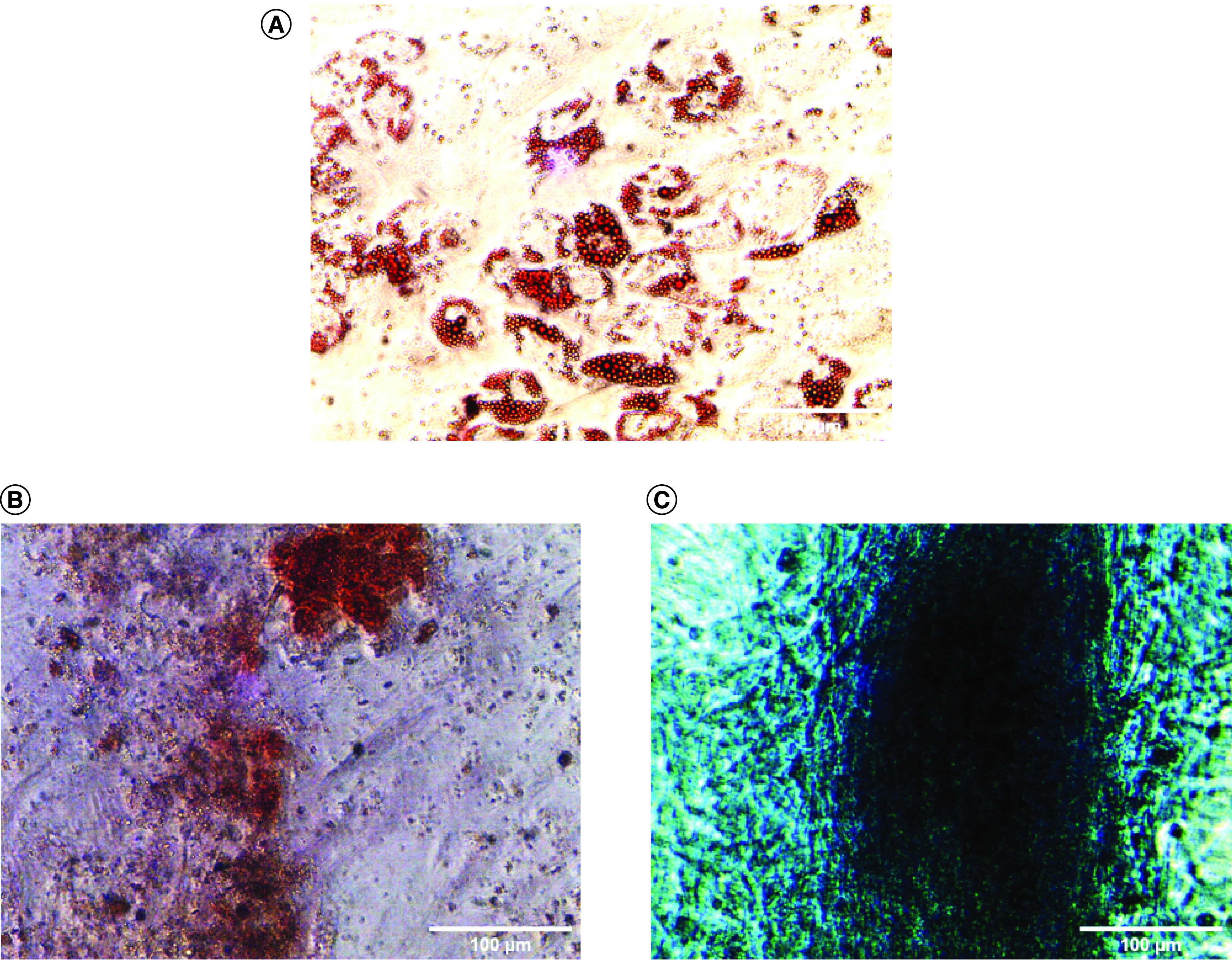
Multipotent differentiation of human adipose-derived stem cells *in vitro*. **(A)** Oil Red O staining showed positive result of adipogenic differentiation detected by lipid droplets. **(B)** Alizarin Red staining showed positive result of osteogenic differentiation. **(C)** Alcian Blue staining shows a positive result of condrogenic differentiation.

The results of the cell isolation and adhesion characterization can be seen in Figure 1. Observation of the cells using an inverted microscope showed that the cells were spindle shaped resembling fibroblasts and adhered to the base of the polystyrene substrate 3 h after seeding. This showed that the cells were adherent. Flourescene-activated cell sorting analysis of the cells showed that 91.97% of the cells expressed CD90, 99.87% expressed CD73, 66.9% expressed CD105 and 0.33% were Lin-negative (Figure 2). The positive values for CD 90, CD 73 and CD 105 and Lin-negative fulfill the minimal requirements of surface markers for MSCs. Multipotency analysis were seen from the staining of cells induced in induction media. Isolated cells grown in adipogenic induction medium could differentiate into adipocytes as shown by the formation of lipid droplets stained by Oil Red (Figure 3A). Staining with Alizarin Red showed that the cell culture could differentiate into osteoblast shown by the presence of calcium mineralization. Alizarin Red stained calcium to become red in color (Figure 3B). The cell had differentiated into chondrocyte indicated by proteoglycan in Alcian Blue staining (Figure 3C). These staining results showed that the source of cells had mutipotent capability. Therefore cells obtained from liposuction waste used in this research had the minimum requirement criteria of a MSC according to Dominici *et al.* [28] presented at The International Society for Cellular Therapy (ISCT) and the cells can be named hADSC.

### Cell penetration on the scaffold

**Figure 4.**
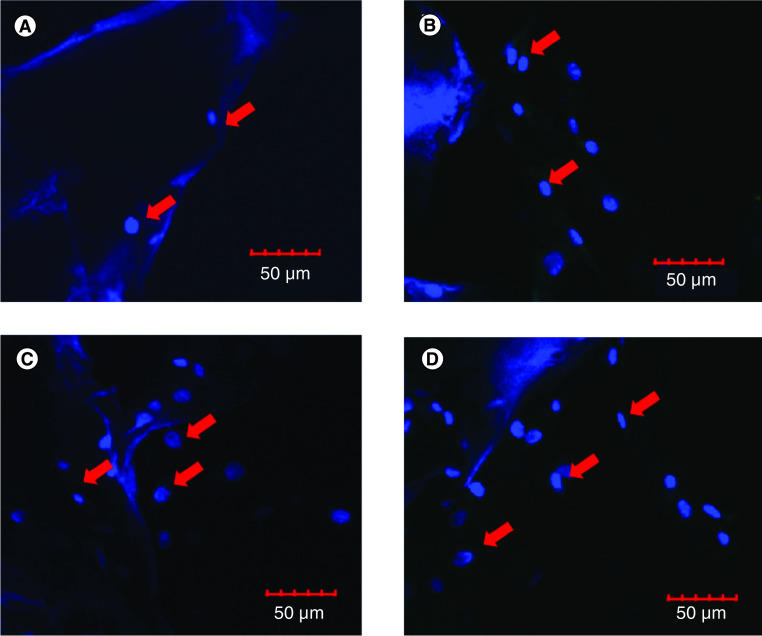
Visualization of cell penetration in silk fibroin scaffold by using confocal laser scanning microscopy. **(A)** hADSCs cultured on scaffold for 7 days, upper surface of scaffold. **(B)** hADSCs cultured on scaffold for 21 days, upper surface of scaffold. **(C)** hADSCs cultured on scaffold for 7 days, lower surface of scaffold. **(D)** hADSCs cultured on scaffold for 21 days, lower surface of scaffold. The red arrows indicate nucleus. hADSC: Human adipose-derived stem cell.

Visualization with a confocal microscope showed that on the seventh day hADSC had started to penetrate and proliferate on the scaffold (Figure 4). Since the nuclei and scaffolds were stained blue by DAPI staining, they had to be differentiated based on their morphology. Based on the abundance of nuclei on the lower surface of the scaffold, it could be assumed that the scaffold that was used in this research had a good porosity and interconnectivity that could support it to become a niche for hADSC in cartilage tissue engineering.

### Relative expression of N-cadherin, β-catenin, cyclin D & type II collagen genes during hADSC chondrogenesis grown on 3D silk fibroin scaffold

**Figure 5.**
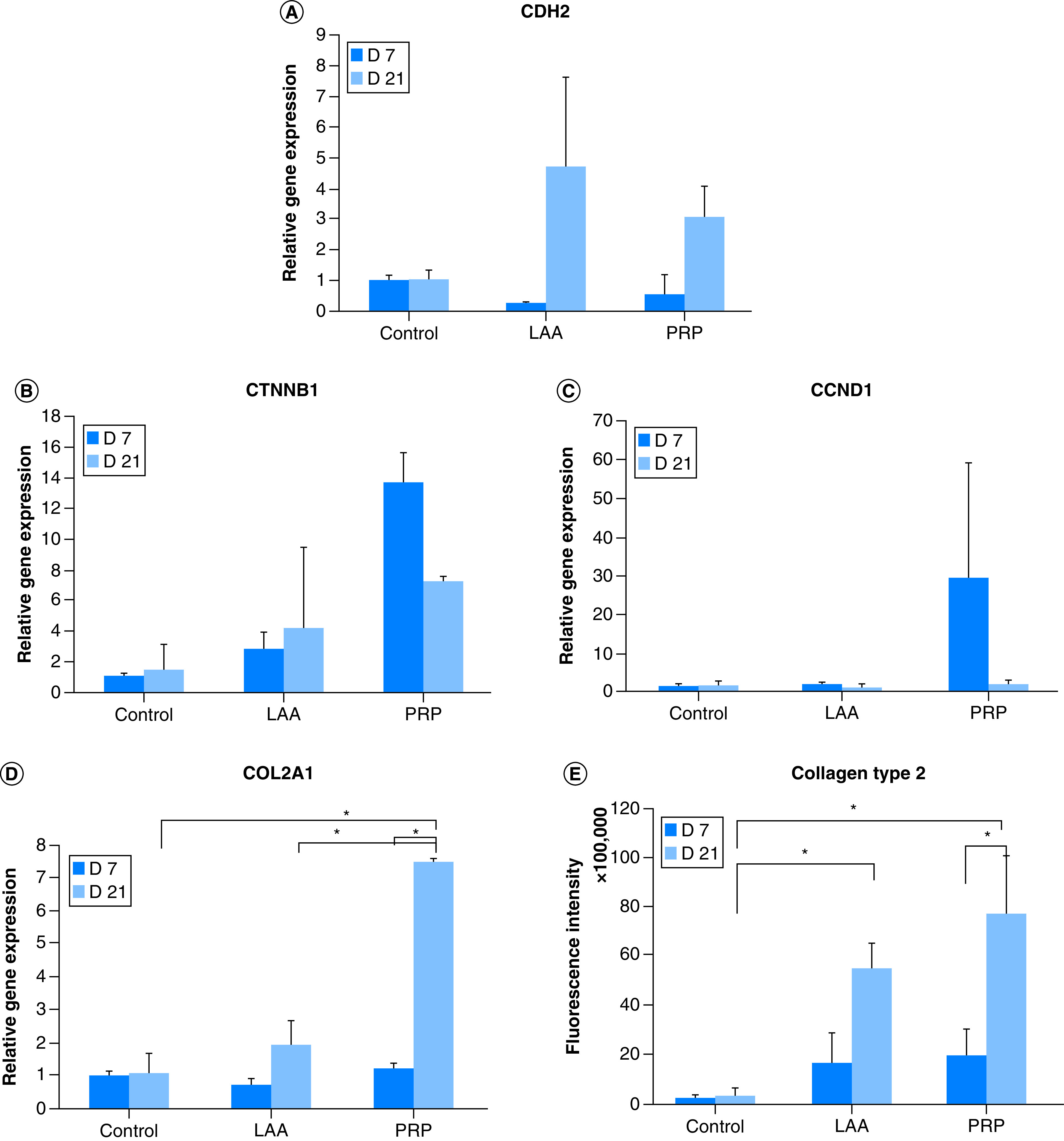
Reverse transcription-quantitative PCR analysis and type 2 collagen fluorescence quantification of control, L-ascorbic acid and platelet rich plasma treatment. **(A)** Relative gene expression of *CDH2* (N cadherin). **(B)** Relative gene expression of *CTNNB1* (β-catenin). **(C)** Relative gene expression of *CCND1* (cyclin D1). **(D)** Relative gene expression of *COL2A1* (type 2 collagen). **(E)** Fluorescence quantification of type 2 collagen. *p < 0.05. LAA: L-ascorbic acid; PRP: Platelet rich plasma.

On the 21st day of culture, the level of expression of *CDH2* gene during hADSC culture treated with 50 μg/ml LAA and PRP 10% that were grown on the silk fibroin scaffold was higher compared with the control but there was no significant difference (Figure 5). The treatment of PRP enhanced the expression of *CTNNB1* gene (β-catenin) compared with control on 7th day of treatment but it suppressed the gene in long period (day 21). The analysis of *CCND1* (Cyclin D) relative gene expression showed that there was no significant difference in the expression of *CCND1* gene among treatments. The analysis of the *COL2A1* relative gene expression showed that treatment with PRP for 21 days increased the expression compared with control.

### Presence of the type II collagen matrix

**Figure 6.**
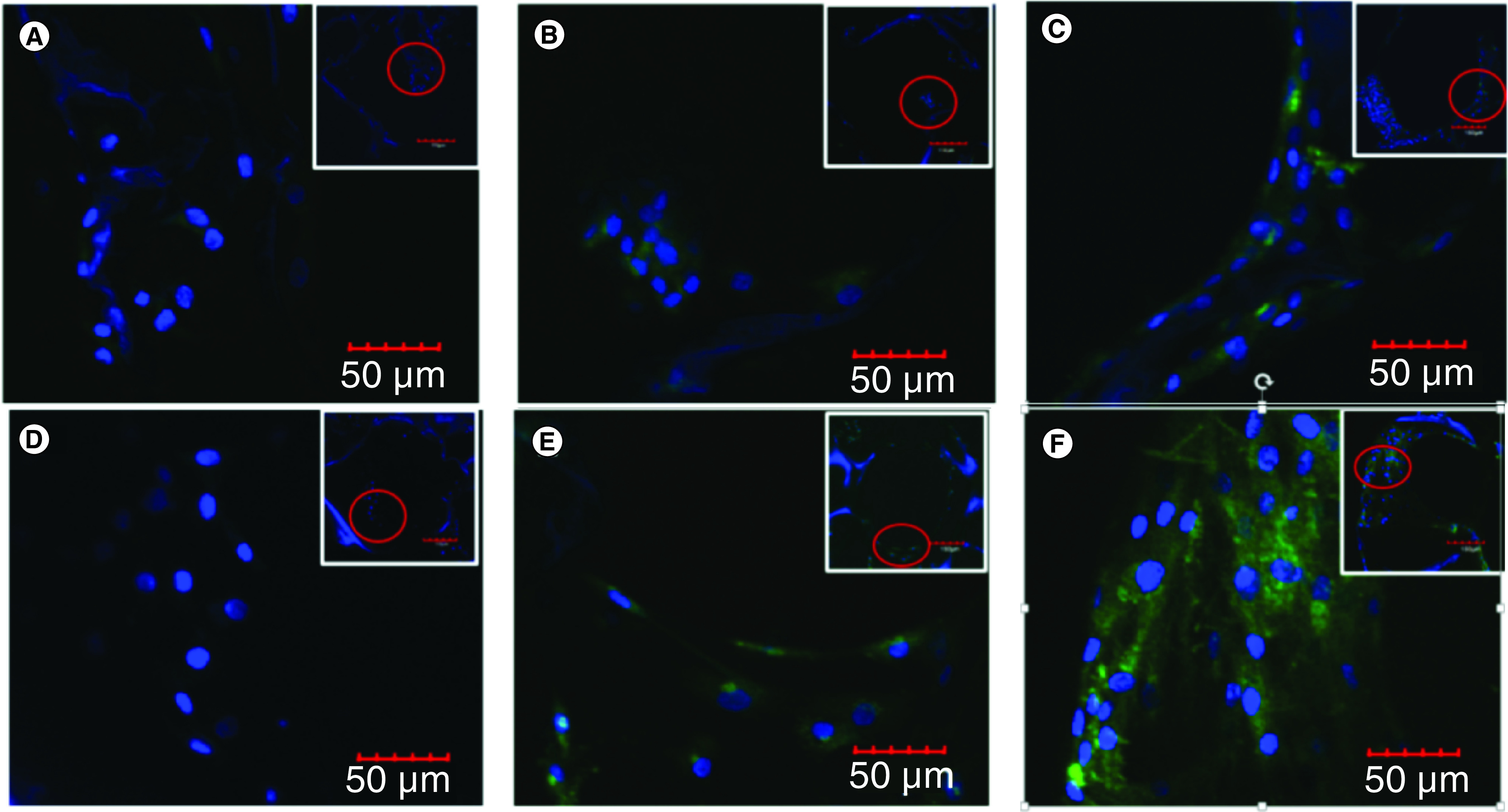
The presence of type 2 collagen matrix of control, L-ascorbic acid and platelet rich plasma treatment. **(A)** Control was cultured for 7 days. **(B)** LAA treatment was cultured for 7 days. **(C)** PRP treatment was cultured for 7 days. **(D)** Control was cultured for 21 days. **(E)** LAA treatment was cultured for 21 days. **(F)** PRP treatment was cultured for 21 days. LAA: L-ascorbic acid; PRP: Platelet rich plasma.

To confirm whether the expression of *COL2A1* gene at the mRNA level correlated with the expressed protein, an immunohistochemical analysis was performed. Based on the quantification of the intensity of type II collagen fluorescence, it could be seen that the expressed type II collagen protein correlated with transcription of *COL2A1* gene (Figure 6 & Figure 5E). In the 50 μg/ml LAA treatment, the amount of type II collagen on the 21st day was higher than the 7th day based on the fluorescence intensity of the labeled type II collagen. In the 10% PRP treatment, the amount of type II collagen was also higher on the 21st day compared with the 7th day.

## Discussion

Success of tissue engineering is depend on the availability of progenitor cells, 3D biomaterial that supports cell growth and differentiation and a source of regulatory signals that are affordable and effective. Previous study of the development of cartilage tissue engineering has used the combination of scaffold with the cell source from amniotic fluid stem cells [29], bone marrow stem cells [30] and adipose stem cells [31]. In this research the cell source used was hADSCs from liposuction because they have the potential for allogeneic cell therapy due to low immunogenic profile [32], abundance in cell number [7], and are a waste product of liposuction. These progenitor cells fulfill the criteria of MSCs; they were adherent, expressed positive surface markers for CD 105, CD 75, CD 90 and had the capability to differentiate into chondrocytes, osteocytes and adipocytes. Barlian *et al.* [26] showed that the silk fibroin scaffold could facilitate the process of cell proliferation and differentiation. Another study using Microcomputed tomography (micro-CT) verified that silk fibroin scaffold was successfully fabricated using 12% w/v silk fibroin and produced 500 μm pores [33]. In this study, the 12% silk fibroin scaffold with 500 μm pores were confirmed to be able to facilitate the penetration of the cells into the scaffold. Cell penetration was important to ensure the suitability of the silk fibroin scaffold in supporting cartilage tissue engineering. The cells were able to penetrate from the upper surface of the scaffold to the lower surface of the scaffold (Figure 4). One of the factors that affected this penetration was the microstructure of the scaffold (porosity and pore size). Our previous studies characterized the microstructure of the scaffold and showed that the scaffold has good interconnectivity pores [11,33]. According to O’Brien [34], the size of the pores had to be sufficiently large to facilitate cell migration and nutrient transport in the scaffold, however it should not be too large that the specific surface area become too small and limited the number of cells that could adhere.

Regulatory signals in the form of LAA and PRP could be used as an alternative that supports cartilage tissue engineering. The effects of LAA and PRP on genes involved in proliferation and differentiation is not yet known. *CDH2* (N-cadherin), *CTNNB1* (β-catenin), *CCND1* (cyclin D) and *COL2A1* (type II collagen) genes were genes involved in the Wnt/β-catenin signaling pathway that played a role in the proliferation and differentiation steps. The expression levels of *CDH2* (N-cadherin), *CTNNB1* (β-catenin), *CCND1* (cyclin D1) and *COL2A1* (type II collagen) genes were interrelated. N-cadherin could inhibit the activity of β-catenin which was a regulator for the Wnt/β-catenin signaling pathway. In this research PRP could inhibit the relative expression of β-catenin and cyclin D genes on the 21st day of treatment, on the other hand N-cadherin gene expression tended to increase. High levels of N-cadherin would bind β-catenin and would inactivate the Wnt/β-catenin signaling pathway. Low levels of β-catenin gene expression correlated with cyclin D (one of the genes in cell proliferation) gene expression and increased the expression of type II collagen gene. Therefore on the 21st day of treatment, hADSC had entered the differentiation stage to become chondrocyte and probably through the inhibition of β-catenin.

The mechanism of PRP and LAA in regulating these three genes were probably different. PRP could not enter the cell but functioned as a ligand. On the other hand, according to Kim *et al.* [12] LAA could enter the cell through its transporter, sodium-dependent vitamin C transporter (SVCT).

Type II collagen was one of the markers for chondrocytes. Both LAA and PRP could increase the expression of type II collagen on the 21st day of treatment. This meant that LAA and PRP supported the chondrogenic differentiation process by activating the *COL2A1* gene that coded for type II collagen matrix. The presence of the type II collagen matrix was confirmed by immunohistochemistry through Z-stacking. Based on the quantification of the fluorescence intensity of type II collagen, it could be seen that type II collagen protein levels correlated with *COL2A1* mRNA levels. The effect of LAA on the presence of type II collagen matrix has also been shown previously by Choi *et al.* [15], who showed an increase in collagen synthesis by ascorbic acid 2 phosphate in MSC culture during 21 days of treatment.

*In vitro* and *in vivo* studies showed that PRP combined with chondrocyte micrografts was successful for cartilage regeneration in patients with cartilage tissue defects [35,36]. In this research the 10% PRP treatment induced type II collagen. The presence of type II collagen induced by PRP on the 21st day was higher than on the 7th day. PRP contained several growth factors that supported the proliferation and differentiation activity of MSCs into chondrocytes, such as TGF-β, PDGF, IGF and bFGF [37]. The growth factors in PRP functioned as ligands that bind to receptors and inhibited the expression of β-catenin, probably by inactivating Wnt/β-catenin signaling during the 21 days of treatment, which then prevented β-catenin from entering the nucleus and resulted in COL2A1 gene expression to produce type II collagen. According to Luo *et al.* [38], inactivation of the Wnt/β-catenin signaling on hADSCs was necessary for the chondrogenic differentiation. Therefore the decrease of β-catenin expression on the 21st day of treatment lead hADSCs to differentiate into chondrocytes.

This study suggests that the combination of silk fibroin scaffold, hADSCs, and LAA or PRP can be beneficial for cartilage tissue engineering. However, further experiments need to be conducted to confirm the stability of the cartilage using those combinations in the *in vivo* study.

## Conclusion

hADSCs could penetrate the 12% silk fibroin scaffold with 500 μm pore size from the top to the bottom of the scaffold. The 50 μg/ml LAA and 10% PRP increased the expression of N-cadherin (*CDH2*) on the 12% silk fibroin scaffold with 500 μm pore size albeit not significantly. The 50 μg/ml LAA and 10% PRP affected the Wnt/β-catenin signaling pathway through the expressions of β-catenin (*CTNNB1*), cyclin D (*CCND1*) and type II collagen (*COL2A1*) genes in the hADSC on the silk fibroin scaffold. The 50 μg/ml LAA and 10% PRP affected the synthesis of type II collagen matrix by hADSCs which differentiated into chondrocytes on the 12% silk fibroin scaffold with 500 μm pore size. The 10% PRP treatment could produce a higher concentration of type II collagen compared with the 50 μg/ml LAA treatment.

## Future perspective

Cartilage has low regeneration capability because of a lack of blood supply. The main goal of cartilage engineering is to make functional cartilage construct to regenerate or repair damaged tissue. The current challenge in cartilage engineering is determining its ideal components such as cell source, the biomaterial and regulatory signal. The combination of tissue engineering in this research: hADSC as a promising cell source, silk fibroin scaffold as a biomaterial, and LAA or PRP as the chemical signal, could promote the differentiation of stem cells into chondrocytes. It also provides clues to the development of tissue engineering and stem cell therapy to overcome cartilage damages in the future. LAA and PRP can be a good regulatory signal to improve stem cell differentiation to make cartilage. The right components can produce functional cartilage and successful treatment of diseases.

**Executive summary**

- The differentiation of human adipose-derived stem cell (hADSC) in silk fibroin scaffold with the addition of L-ascorbic acid (LAA) or platelet rich plasma (PRP) were observed.
- Z-stack confocal microscopy analysis showed that hADSCs can penetrate into silk fibroin scaffold with 500 μm pore size.
- LAA and PRP affect the relative expression of differentiation genes in Wnt/β catenin pathway.
- hADSCs can differentiate into chondrocytes in silk fibroin scaffold.
- The extracellular matrix of chondrocytes, type II collagen, was present in the scaffold seeded with hADSCs and supplemented with LAA.
- PRP induces the production of type II collagen better than LAA.
