# Supplementary material for: Cell penetration and chondrogenic differentiation of human adipose derived stem cells on 3D scaffold
Source: Future Sci OA. 2021 Jun 10;7(8):FSO734. doi: 10.2144/fsoa-2021-0040 (PMC8288224; doi:10.2144/fsoa-2021-0040)
Supplement: Supplementary file 1 [file fsoa-07-734-s1.docx]

Supplementary Table 1. List of primers used for RT-qPCR

| Gen | NCBI ref seq | Primer | Sekuen |
| --- | --- | --- | --- |
| *Cdh2* | NM_001308176.1 | *Forward* | GACCCAAACAGCAACGACGG |
|  |  | *Reverse* | GCGGGTGCTGAATTCCCTTG |
| *Col2a1* | NM_001844.4 | *Forward* | GAACCCAGAAACAACACAATCC [39] |
|  |  | *Reverse* | CATTCAGTGCAGAGTCCTAGAG [39] |
| *Ccnd1* | NM_053056.2 | *Forward* | GGAGCCCGTGAAAAAGAGCC |
|  |  | *Reverse* | TCATTGCGGCCAGGTTCCAC |
| *Ctnnb1* | NM_001904.3 | *Forward* | ACATCAGGATACCCAGCGCC |
|  |  | *Reverse* | GGGCTCCGGTACAACCTTCA |
| *Gapdh* | NM_002046.6 | *Forward* | TCCTGTTCGACAGTCAGCCG |
|  |  | *Reverse* | CCCCATGGTGTCTGAGCGAT |
